# Monitoring Influenza Activity in the United States: A Comparison of Traditional Surveillance Systems with Google Flu Trends

**DOI:** 10.1371/journal.pone.0018687

**Published:** 2011-04-27

**Authors:** Justin R. Ortiz, Hong Zhou, David K. Shay, Kathleen M. Neuzil, Ashley L. Fowlkes, Christopher H. Goss

**Affiliations:** 1 University of Washington, Seattle, Washington, United States of America; 2 Centers for Disease Control and Prevention, Atlanta, Georgia, United States of America; 3 Atlanta Research and Education Foundation, Atlanta, Georgia, United States of America; 4 PATH, Seattle, Washington, United States of America; National University of Singapore, Singapore

## Abstract

**Background:**

Google Flu Trends was developed to estimate US influenza-like illness (ILI) rates from internet searches; however ILI does not necessarily correlate with actual influenza virus infections.

**Methods and Findings:**

Influenza activity data from 2003–04 through 2007–08 were obtained from three US surveillance systems: Google Flu Trends, CDC Outpatient ILI Surveillance Network (CDC ILI Surveillance), and US Influenza Virologic Surveillance System (CDC Virus Surveillance). Pearson's correlation coefficients with 95% confidence intervals (95% CI) were calculated to compare surveillance data. An analysis was performed to investigate outlier observations and determine the extent to which they affected the correlations between surveillance data. Pearson's correlation coefficient describing Google Flu Trends and CDC Virus Surveillance over the study period was 0.72 (95% CI: 0.64, 0.79). The correlation between CDC ILI Surveillance and CDC Virus Surveillance over the same period was 0.85 (95% CI: 0.81, 0.89). Most of the outlier observations in both comparisons were from the 2003–04 influenza season. Exclusion of the outlier observations did not substantially improve the correlation between Google Flu Trends and CDC Virus Surveillance (0.82; 95% CI: 0.76, 0.87) or CDC ILI Surveillance and CDC Virus Surveillance (0.86; 95%CI: 0.82, 0.90).

**Conclusions:**

This analysis demonstrates that while Google Flu Trends is highly correlated with rates of ILI, it has a lower correlation with surveillance for laboratory-confirmed influenza. Most of the outlier observations occurred during the 2003–04 influenza season that was characterized by early and intense influenza activity, which potentially altered health care seeking behavior, physician testing practices, and internet search behavior.

## Introduction

The emergence of 2009 pandemic influenza A (H1N1) virus in the United States and Mexico, and its subsequent rapid global spread has underscored the importance of influenza surveillance for public health decision making [1]. Recently, Google.org developed Google Flu Trends, a model to estimate US influenza-like illness (ILI) rates from internet searches. The model was fit to CDC sentinel provider surveillance data for ILI from 2003 to 2007 and prospectively validated using the same surveillance system during the 2007–08 influenza season. During the 2007–08 influenza season, Google Flu Trends estimates were highly correlated to CDC surveillance for ILI, with a mean correlation coefficient over nine US Census Regions of 0.97 [2].

For the purpose of CDC influenza surveillance, ILI is defined as a fever ≥37.8°C and a cough and/or a sore throat without known etiology (http://www.cdc.gov/flu/weekly/fluactivity.htm, accessed 09/04/09). ILI is not specific to influenza, however. Prospective studies with laboratory sampling of persons with ILI have demonstrated a wide variability in the specificity of ILI for influenza disease, with the proportion of subjects testing positive for influenza ranging from 20% to 70% of those tested during the influenza season [3], [4]. ILI may also not be sensitive for influenza, particularly in certain age or risk groups where influenza may have atypical presentations [5], [6], [7], [8], [9]. Furthermore, even during peak periods of influenza circulation, a substantial number of cases of febrile respiratory illness may have non-influenza etiologies. In the United States, during the spring wave of the 2009 H1N1 outbreak from March through August 2009, the proportion of positive influenza laboratory tests did not exceed 45% (http://www.cdc.gov/flu/weekly, accessed 09/04/09).

Because Google Flu Trends estimates of ILI may not necessarily correlate with actual influenza virus infections, we undertook this study to evaluate how Google Flu Trends influenza surveillance data compared with national surveillance data for laboratory-confirmed influenza infections.

## Methods

National and regional estimates of the weekly percentage of persons seeking health care in the United States with ILI were obtained from Google Flu Trends on June 11, 2009 (http://www.google.org/about/flutrends/us-historic.txt). The remaining surveillance data were obtained from CDC. These data were from two separate surveillance networks: Outpatient Influenza-like Illness Surveillance Network (CDC ILI Surveillance) and the US Influenza Virologic Surveillance System (CDC Virus Surveillance). CDC ILI Surveillance consists of a network of health care providers who record the weekly proportion of patients who present with non-specific signs and symptoms that meet a case definition of influenza-like illness [10]. CDC Virus Surveillance consists of about 140 laboratories located throughout the United States that report the weekly total specimens tested and laboratory tests positive for influenza virus [10]. This is the only US surveillance system that provides national and regional data of laboratory-confirmed influenza virus infection. CDC Virus Surveillance is used in CDC statistical models in the estimation of influenza-associated morbidity and mortality [11], [12], [13], [14], [15], [16]. For this analysis, CDC Influenza Virus Surveillance was used as the reference standard to which Google Flu Trends and CDC ILI Surveillance were compared.

The study period was September 28, 2003 through May 17, 2008. These dates were chosen to include all available Google Flu Trends historical ILI estimates and exclude the 2009 H1N1 pandemic which began during the 2008–09 influenza season. Analyses were performed by “influenza season,” defined as the period from July 1 through June 30 of the subsequent calendar year. As done in similar analyses [2], we restricted our analysis to the period during which CDC influenza surveillance is intensified, from calendar week 40 through calendar week 20 of the subsequent year.

For the primary analysis, scatter plots with least square regression lines were constructed to compare Google Flu Trends and CDC ILI Surveillance to the standard reference surveillance (CDC Virus Surveillance). Pearson's correlation coefficients with 95% confidence intervals (95% CI) were then computed from these comparisons. Subsequently, additional correlation coefficients with 95% CI were calculated from surveillance comparisons by influenza season, US Census Region, and influenza season categorized by US Census Region. These subset analyses were summarized with mean correlation coefficients and standard deviations (SD). Next, because Flu Trends was previously found to lead CDC ILI Surveillance observations by one to two weeks [2], we undertook additional correlation analyses to determine whether Google Flu Trends or CDC ILI Surveillance had a stronger correlation with CDC Virus surveillance data for the subsequent one or two weeks. The unit of analysis was percentage of clinic patients with ILI (CDC ILI Surveillance and Google Flu Trends) and percentage of laboratory tests positive for influenza (CDC Virus Surveillance).

To ensure this assessment of surveillance data was comparable to the study that validated Google Flu Trends [2], we performed a secondary analysis replicating methods from that study with our dataset. The mean coefficient of correlation between Google Flu Trends and CDC ILI Surveillance was calculated over nine US Census Regions for the 2007–08 influenza season. The mean coefficient of correlation between Google Flu Trends and CDC Virus Surveillance was calculated similarly for comparison.

We also performed a secondary analysis to determine the sensitivity of the primary analysis to high-leverage, outlier observations. First, we performed simple linear regression to evaluate the association between either Google Flu Trends or CDC ILI Surveillance rates with reference viral surveillance data as standard rates. The effect of outlier observations was assessed with differences in the beta statistic (DFBETA). Individual observations were considered influential if they had a DFBETA greater than the absolute value of 2 divided by the square root of the total number of observations in the model [17]. Subsequently, all influential observations were excluded and correlation coefficients were recalculated as had been done in the primary analysis. Correlation coefficients from the sensitivity analysis were compared to the same statistic from the primary analysis to determine whether any relevant changes in the strength of correlation had occurred with the removal of influential observations. Last, Spearman Rank correlation coefficients were employed for the primary analyses and noted to yield similar results.

This study received exempt review status from the Human Subjects Division at the University of Washington. Analyses were performed with STATA statistical software (version 10.1; STATA Corporation; College Station, TX).

## Results

**Figure 1:**
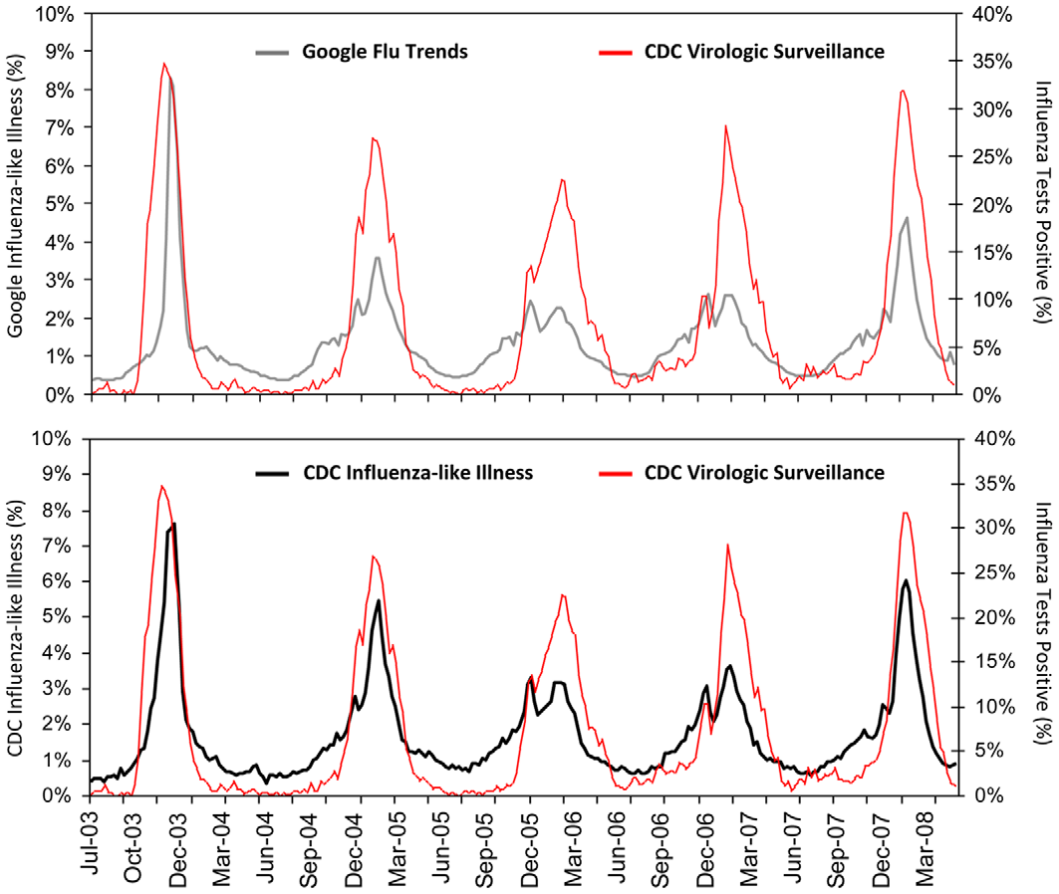
United States Influenza Surveillance by Google Flu Trends^1^, CDC Influenza-like Illness Surveillance^2^, and CDC Influenza Virologic Surveillance^3^, June 29, 2003 through May 31, 2008^4^. ^1^Google Flu Trends estimates the percentage of persons seeking health care for the non-specific complaint of influenza-like illness (ILI) based on internet key word searches. ^2^CDC Influenza-like Illness Surveillance involves a network of health care providers who record the weekly proportion of patients seen with ILI. Google Flu Trends was created and validated using CDC ILI Surveillance data, explaining the similarity between the two curves. ^3^CDC Influenza Virologic Surveillance consists of about 140 laboratories located throughout the United States that report the weekly total specimens tested and laboratory tests positive for influenza virus. This is the only US surveillance system that provides national and regional data of laboratory-confirmed influenza virus infection. ^4^Because CDC surveillance is intensified from calendar week 40 through calendar week 20 of the subsequent year, we restricted our correlation analyses to this time period.

**Figure 2:**
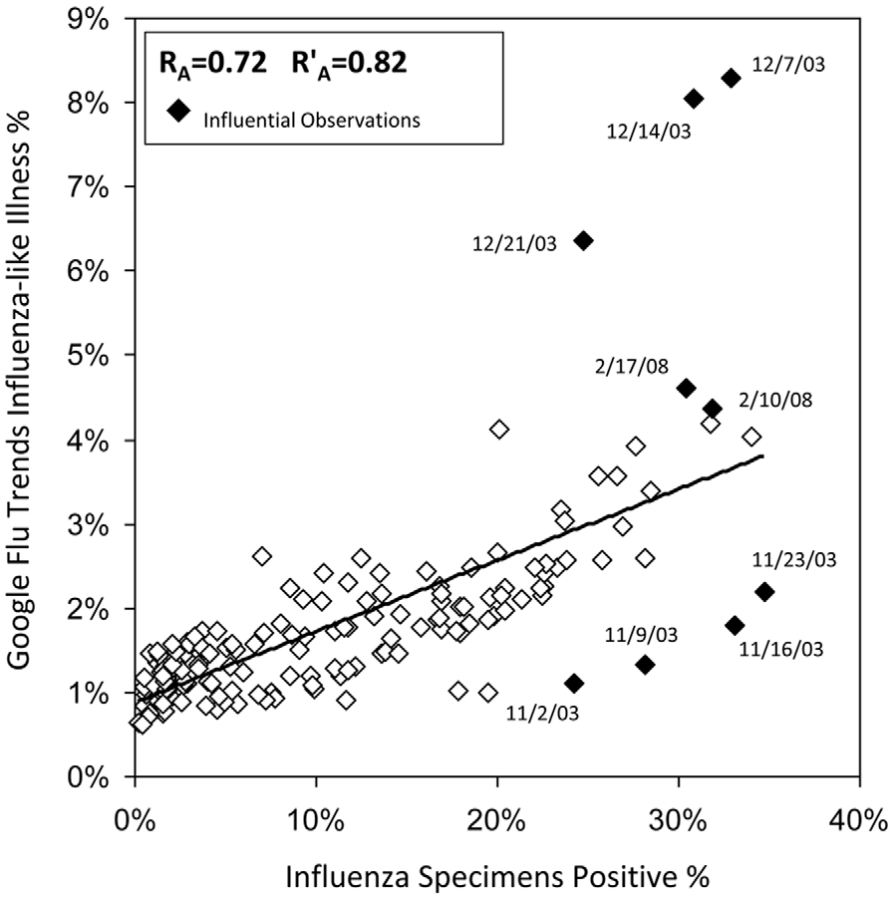
Scatter Plot Google Flu Trends and CDC Influenza Laboratory Surveillance; September 28, 2003 through May 17, 2008. **1.** Data Sources: **a.** US Influenza Virologic Surveillance System (http://www.cdc.gov/flu/weekly/fluactivity.htm); and **b.** Google Flu Trends (http://www.google.org/about/flutrends/us-historic.txt). **2.** There are 166 total observations in each panel. Influential observations were defined by DFBETA statistic greater than the absolute value of 2 divided by the square root of the total number of observations in a simple linear regression model [17]. **3.** R_A_ represents Pearson's correlation coefficients calculated from comparisons of Google Flu Trends with US Influenza Virologic Surveillance. **4.** R'_A_ represents the calculated Pearson's correlation coefficients after exclusion of all influential observations. **5.** Because CDC surveillance is intensified from calendar week 40 through calendar week 20 of the subsequent year, we restricted our correlation analyses to this time period.

**Figure 3:**
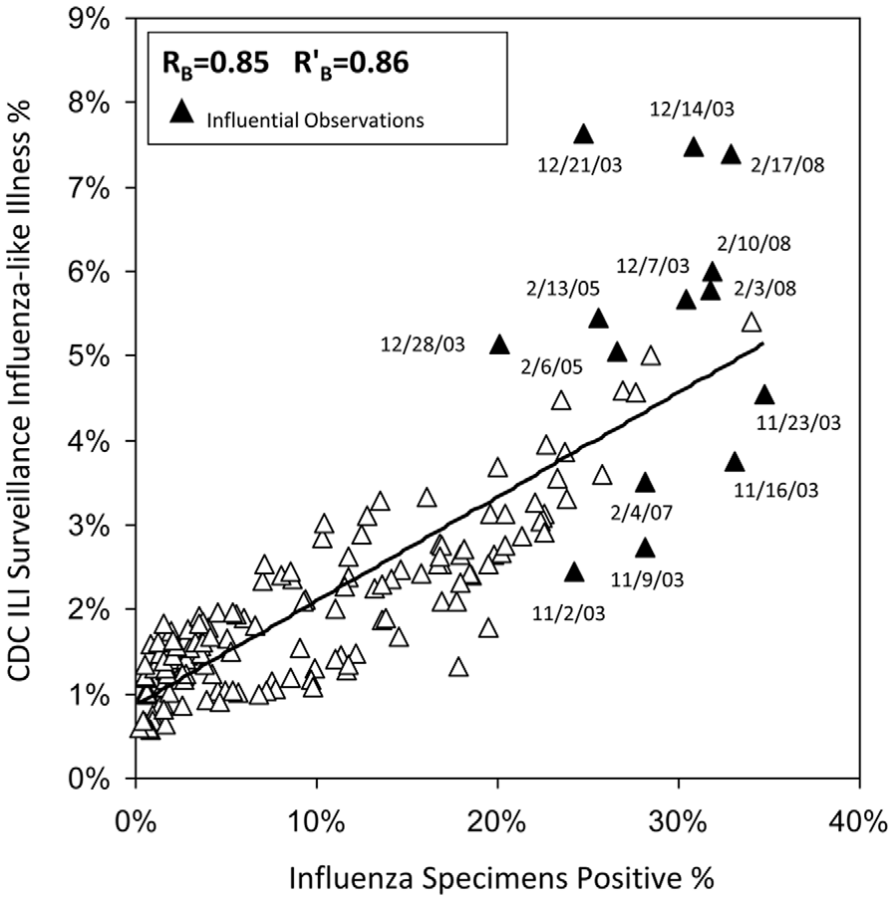
Scatter Plot CDC ILI Surveillance and CDC Influenza Laboratory Surveillance; September 28, 2003 through May 17, 2008. **1.** Data Sources: **a.** Outpatient Influenza-like Illness Surveillance Network; (http://www.cdc.gov/flu/weekly/fluactivity.htm); and **b.** US Influenza Virologic Surveillance System (http://www.cdc.gov/flu/weekly/fluactivity.htm). **2.** For CDC influenza surveillance, Influenza-like Illness (ILI) is defined as a fever ≥37.8°C and a cough and/or a sore throat without known etiology [22]. **3.** There are 166 total observations in each panel. Influential observations were defined by DFBETA statistic greater than the absolute value of 2 divided by the square root of the total number of observations in a simple linear regression model [17]. **4.** R_B_ represents Pearson's correlation coefficients calculated from comparisons of Outpatient Influenza-like Illness Surveillance with US Influenza Virologic Surveillance. **5.** R'_B_ represents the calculated Pearson's correlation coefficients after exclusion of all influential observations. **6.** Because CDC surveillance is intensified from calendar week 40 through calendar week 20 of the subsequent year, we restricted our correlation analyses to this time period.

Our analyses used 166 weeks of data from the 2003–04 through the 2007–08 influenza seasons obtained from three influenza surveillance systems used to monitor national and regional influenza trends. Data included five influenza seasons from 2003–04 through the 2007–08 influenza season. There was a strong temporal association among rates from each surveillance system (Figure 1). Scatter plots of Google Flu Trends and CDC ILI Surveillance with the reference standard data showed high linear correlations (Figure 2 and Figure 3). Pearson's correlation coefficient describing the strength of association between Google Flu Trends with CDC Virus Surveillance was 0.72 (95% CI: 0.64, 0.79) (Figure 2 and Table 1). The strength of association between CDC ILI Surveillance and CDC Virus Surveillance was higher, with a correlation coefficient of 0.85 (95% CI: 0.81, 0.89) (Figure 3 and Table 1). Google Flu Trends, which had been fit to CDC ILI Surveillance, was highly correlated to that surveillance data (R = 0.94; 95%CI: 0.92, 0.96).

**Table 1:** Pearson's Correlation Coefficient Matrix of Data from Three Influenza Surveillance Systems: Google Flu Trends, CDC Influenza-like Illness Surveillance, CDC Influenza Virologic Surveillance, September 28, 2003 through May 17, 2008^1^.

Dataset	Google Flu Trends	CDC ILI	CDC Virologic
Google Flu Trends	1.00	--	--
CDC ILI	0.94 (0.92, 0.96)	1.00	--
CDC Virologic	0.72 (0.64, 0.79)	0.85 (0.81, 0.89)	1.00
CDC Virologic plus One Week	0.69 (0.60, 0.76)	0.79 CI: 0.72, 0.84	--
CDC Virologic plus Two Weeks	0.66 (0.56, 0.74)	0.75 (0.68, 0.81)	--

1 Because CDC surveillance is intensified from calendar week 40 through calendar week 20 of the subsequent year, we restricted our correlation analyses to this time period.

### Annual Analysis

Correlations among influenza surveillance systems differed by influenza season. The correlation coefficient describing the association between Google Flu Trends and CDC Virus surveillance ranged from 0.67 (95% CI 0.43, 0.82) during the 2003–04 influenza season to 0.94 (95% CI 0.89, 0.97) during the 2004–05 influenza season (Table 2). The mean correlation coefficient for these comparisons was 0.79 (SD 0.13). The correlation between CDC ILI Surveillance and CDC Virus Surveillance ranged from 0.79 (95% CI 0.62, 0.89) during the 2005–06 influenza season to 0.94 (95% CI 0.88, 0.97) during the 2004–05 influenza season. The mean correlation coefficient for these comparisons was 0.86 (SD 0.07).

**Table 2:** Pearson's Correlation Coefficient Matrix of Data from Three Influenza Surveillance Systems by Surveillance Year: Google Flu Trends, CDC Influenza-like Illness Surveillance, CDC Influenza Virologic Surveillance, September 28, 2003 through May 17, 20081.

Surveillance Year	Dataset	Google Flu Trends	CDC ILI	CDC Virologic
2003–04	Google Flu Trends	1.00	--	--
	CDC ILI	0.94 (0.89, 0.97)	1.00	--
	CDC Virologic	0.67 (0.43, 0.82)	0.84 (0.69, 0.92)	1.00
2004–05	Google Flu Trends	1.00	--	--
	CDC ILI	0.98 (0.95, 0.99)	1.00	--
	CDC Virologic	0.94 (0.89, 0.97)	0.94 (0.88, 0.97)	1.00
2005–06	Google Flu Trends	1.00	--	--
	CDC ILI	0.97 (0.94, 0.99)	1.00	--
	CDC Virologic	0.72 (0.50, 0.85)	0.79 (0.62, 0.89)	1.00
2006–07	Google Flu Trends	1.00	--	--
	CDC ILI	0.94 (0.89, 0.97)	1.00	--
	CDC Virologic	0.71 (0.49, 0.85)	0.81 (0.64, 0.90)	1.00
2007–08	Google Flu Trends	1.00	--	--
	CDC ILI	0.98 (0.96, 0.99)	1.00	--
	CDC Virologic	0.91 (0.82, 0.95)	0.92 (0.85, 0.96)	1.00

1 Because CDC surveillance is intensified from calendar week 40 through calendar week 20 of the subsequent year, we restricted our correlation analyses to this time period.

### Regional Analysis

Correlations among influenza surveillance systems also differed by US Census Region. The correlation coefficients describing the association between Google Flu Trends and CDC Virus Surveillance over the study period ranged from 0.64 (95% CI 0.54, 0.72) in the East North Central Region to 0.80 (95% CI 0.74, 0.85) in the West North Central Region (Table 3). The mean correlation coefficient for these comparisons was 0.70 (SD 0.05). The correlation between CDC ILI Surveillance and CDC Virus Surveillance ranged from 0.64 (95% CI 0.55, 0.73) in the East South Central Region to 0.86 (95% CI 0.81, 0.89) in the West South Central Region. The mean correlation coefficient for these comparisons was 0.76 (SD 0.07).

**Table 3:** Pearson's Correlation Coefficient Matrix of Data from Three Influenza Surveillance Systems by US Census Region: Google Flu Trends, CDC Influenza-like Illness Surveillance, CDC Influenza Virologic Surveillance, September 28, 2003 through May 17, 20081.

US Census Region^2^	Dataset	Google Flu Trends	CDC ILI	CDC Virologic
New England	Google Flu Trends	1.00	--	--
	CDC ILI	0.94 (0.92, 0.96)	1.00	
	CDC Virologic	0.65 (0.55, 0.73)	0.76 (0.68, 0.82)	1.00
Middle Atlantic	Google Flu Trends	1.00	--	--
	CDC ILI	0.87 (0.82, 0.90)	1.00	
	CDC Virologic	0.67 (0.58, 0.75)	0.70 (0.61, 0.77)	1.00
East North Central	Google Flu Trends	1.00	--	--
	CDC ILI	0.96 (0.94, 0.97)	1.00	
	CDC Virologic	0.64 (0.54, 0.72)	0.73 (0.65, 0.80)	1.00
West North Central	Google Flu Trends	1.00	--	--
	CDC ILI	0.95 (0.93, 0.97)	1.00	
	CDC Virologic	0.80 (0.74, 0.85)	0.82 (0.76, 0.87)	1.00
South Atlantic	Google Flu Trends	1.00	--	--
	CDC ILI	0.91 (0.88, 0.93)	1.00	--
	CDC Virologic	0.72 (0.64, 0.79)	0.82 (0.76, 0.86)	1.00
East South Central	Google Flu Trends	1.00	--	--
	CDC ILI	0.81 (0.75, 0.86)	1.00	--
	CDC Virologic	0.69 (0.60, 0.76)	0.64 (0.55, 0.73)	1.00
West South Central	Google Flu Trends	1.00	--	--
	CDC ILI	0.87 (0.82, 0.90)	1.00	--
	CDC Virologic	0.74 (0.70, 0.80)	0.86 (0.81, 0.89)	1.00
Mountain	Google Flu Trends	1.00	--	--
	CDC ILI	0.91 (0.88, 0.93)	1.00	--
	CDC Virologic	0.72 (0.64, 0.79)	0.81 (0.75, 0.86)	1.00
Pacific	Google Flu Trends	1.00	--	--
	CDC ILI	0.84 (0.79, 0.88)	1.00	--
	CDC Virologic	0.67 (0.58, 0.75)	0.78 (0.71, 0.83)	1.00

Note:

1 Because CDC surveillance is intensified from calendar week 40 through calendar week 20 of the subsequent year, we restricted our correlation analyses to this time period.

2 US Census Regions include the following states: (1) New England – Connecticut, Maine, Massachusetts, New Hampshire, Rhode Island, Vermont; (2) Middle Atlantic – New Jersey, New York, Pennsylvania; (3) East North Central – Indiana, Illinois, Michigan, Ohio, Wisconsin; (4) West North Central – Iowa, Kansas, Minnesota, Missouri, Nebraska, North Dakota, South Dakota; (5) South Atlantic – Delaware, District of Columbia, Florida, Georgia, Maryland, North Carolina, South Carolina, Virginia, West Virginia; (6) East South Central – Alabama, Kentucky, Mississippi, Tennessee; (7) West South Central – Arkansas, Louisiana, Oklahoma, Texas; (8) Mountain – Arizona, Colorado, Idaho, New Mexico, Montana, Utah, Nevada, Wyoming; (9) Pacific – Alaska, California, Hawaii, Oregon, Washington.

### Surveillance Correlation with Subsequent Weeks of Tests Positive for Influenza

We assessed whether Google Flu Trends or CDC ILI Surveillance had a higher correlation with diagnostic tests positive for influenza in subsequent weeks (Table 1). Neither nationwide data from Google Flu Trends nor CDC ILI Surveillance was more highly correlated with CDC Virus Surveillance observations from the subsequent week (0.69 CI: 0.60, 0.76; and 0.79 CI: 0.72, 0.84) or those from two weeks in the future (0.66 CI: 0.56, 0.74; and 0.75 CI: 0.68, 0.81).

With the exception of the 2003–04 influenza season, correlation coefficients decreased proportionately when seasonal Google Flu Trends or CDC ILI Surveillance were assessed against seasonal CDC Virus Surveillance for the subsequent one or two weeks (Table S1). The correlation over the 2003–04 influenza season between Google Flu Trends and CDC Virus Surveillance increased from 0.67 to 0.77 when assessed with a one-week lag and 0.82 when assessed with a two-week lag. CDC ILI Surveillance had a stronger increase from 0.80 to 0.89 with a one-week lag and 0.92 with a two-week lag.

The use of regional surveillance comparisons did not result in increased correlation with virologic surveillance of the subsequent one or two weeks. The mean Google Flu Trends correlation with CDC Virus Surveillance was 0.70 (SD 0.05) at baseline, 0.70 (SD 0.07) with a one-week lag, and 0.63 (SD 0.11) with a two-week lag (Table S2). The mean CDC ILI Surveillance correlation with CDC Virus Surveillance decreased from 0.78 (SD 0.04) at baseline to 0.76 (SD 0.08) with a one-week lag and 0.68 (SD 0.11) with a two-week lag. Calendar week of peak influenza activity per influenza surveillance year for each of the three surveillance systems can be found in Table S3.

### 2007–08 Influenza Season Analysis by US Census Region

We replicated the analysis that validated Google Flu Trends by assessing the correlation with regional CDC ILI Surveillance data during the 2007–08 influenza season (Table 4). The mean correlation coefficient by US Census Region during the 2007–08 influenza season was 0.97 (SD 0.02), identical to the previously published result [2]. When regional Google Flu Trends data were compared to CDC Virus Surveillance, the mean correlation coefficient was lower, 0.87 (SD 0.04). Additional seasonal analyses by region demonstrated that the mean Google Flu Trends-CDC Virus Surveillance correlation coefficient was consistently below the mean correlation between CDC ILI Surveillance and the reference standard by US Census Region (Table S4).

**Table 4:** Pearson's Correlation Coefficient Matrix of Three Influenza Surveillance Systems by US Census Region during the 2007-08 Influenza Season^1^,^2^

US Census Region^3^	Dataset	Google Flu Trends	CDC ILI	CDC Virologic
New England	Google Flu Trends	1.00	--	--
	CDC ILI	0.98 (0.95, 0.99)	1.00	--
	CDC Virologic	0.86 (0.74, 0.93)	0.88 (0.77, 0.94)	1.00
Middle Atlantic	Google Flu Trends	1.00	--	--
	CDC ILI	0.97 (0.03, 0.98)	1.00	--
	CDC Virologic	0.84 (0.70, 0.92)	0.91 (0.82, 0.96)	1.00
East North Central	Google Flu Trends	1.00	--	--
	CDC ILI	0.98 (0.96, 0.99)	1.00	--
	CDC Virologic	0.87 (0.75, 0.93)	0.92 (0.83, 0.96)	1.00
West North Central	Google Flu Trends	1.00	--	--
	CDC ILI	0.98 (0.96, 0.99)	1.00	--
	CDC Virologic	0.82 (0.66, 0.91)	0.87 (0.76, 0.94)	1.00
South Atlantic	Google Flu Trends	1.00	--	--
	CDC ILI	0.98 (0.95, 0.99)	1.00	--
	CDC Virologic	0.90 (0.81, 0.95)	0.90 (0.81, 0.95)	1.00
East South Central	Google Flu Trends	1.00	--	--
	CDC ILI	0.98 (0.95, 0.99)	1.00	--
	CDC Virologic	0.85 (0.72, 0.92)	0.89 (0.79, 0.95)	1.00
West South Central	Google Flu Trends	1.00	--	--
	CDC ILI	0.97 (0.94, 0.99)	1.00	--
	CDC Virologic	0.94 (0.88, 0.97)	0.94 (0.89, 0.97)	1.00
Mountain	Google Flu Trends	1.00	--	--
	CDC ILI	0.98 (0.96, 0.98)	1.00	--
	CDC Virologic	0.91 (0.83, 0.96)	0.91 (0.82, 0.95)	1.00
Pacific	Google Flu Trends	1.00	--	--
	CDC ILI	0.92 (0.84, 0.96)	1.00	--
	CDC Virologic	0.88 (0.77, 0.94)	0.77 (0.58, 0.88)	1.00

Note:

1 Because CDC surveillance is intensified from calendar week 40 through calendar week 20 of the subsequent year, we restricted our correlation analyses to this time period.

2 Overall mean Pearson's correlation coefficient by US Census Region during 2007-08 influenza season:

a. Google Flu Trends and CDC ILI Surveillance: 0.97 (0.02)

b. Google Flu Trends and CDC Virologic Surveillance: 0.87 (0.04)

c. CDC ILI Surveillance and CDC Virologic Surveillance: 0.89 (0.05)

3 US Census Regions include the following states: (1) New England; Connecticut, Maine, Massachusetts, New Hampshire, Rhode Island, Vermont; (2) Middle Atlantic; New Jersey, New York, Pennsylvania; (3) East North Central; Indiana, Illinois, Michigan, Ohio, Wisconsin; (4) West North Central; Iowa, Kansas, Minnesota, Missouri, Nebraska, North Dakota, South Dakota; (5) South Atlantic; Delaware, District of Columbia, Florida, Georgia, Maryland, North Carolina, South Carolina, Virginia, West Virginia; (6) East South Central; Alabama, Kentucky, Mississippi, Tennessee; (7) West South Central; Arkansas, Louisiana, Oklahoma, Texas; (8) Mountain; Arizona, Colorado, Idaho, New Mexico, Montana, Utah, Nevada, Wyoming; (9) Pacific; Alaska, California, Hawaii, Oregon, Washington.

### Sensitivity Analysis

A sensitivity analyses was performed to investigate the possible effects of outlier observations and to determine whether the removal of influential outlier observations substantially affected tests of correlation. In the comparison of Google Flu Trends with CDC Virus Surveillance over the entire study period, 9 (5.4%) of the 166 total observations were found to be influential outliers based on DFBETA values (Figure 2). Seven of the 9 (77.8%) influential observations occurred from November through December 2003. The calculated correlation coefficient after exclusion of these observations was 0.82 (95%CI: 0.76, 0.87), a 14% increase from the primary analysis. On the other hand, in the comparison of CDC ILI Surveillance with CDC Virus Surveillance over the entire study period, 8.4% (14 of 166) of influential observations were found (Figure 3). Of these, 8 of the 14 (57.1%) observations were from November through December 2003. The calculated correlation coefficient after exclusion of these observations was 0.86 (95% CI: 0.82, 0.90), a 1% increase from the primary analysis.

## Discussion

We compared data describing the proportion of subjects testing positive each week for influenza with data from CDC's ILI surveillance system and data from Google Flu Trends. A prior analysis compared Google Flu Trends data to US Census Region ILI data and demonstrated a strong correlation during the 2007–08 influenza season (R = 0.97) [2]. The correlation between Google Flu Trends data and national influenza test data was lower (R = 0.72) when assessed over five influenza seasons beginning in 2003. In terms of coefficients of determination (R^2^), 88% of the variance is shared between Google Flu Trends and CDC ILI Surveillance, while only 51% of the variance is shared between Google Flu Trends and surveillance for laboratory-confirmed influenza. From September 2003 through May 2008, CDC ILI surveillance was more closely correlated with CDC Virus Surveillance (R = 0.85). Furthermore, the sensitivity analysis demonstrated that the Google Flu Trends correlation with the reference standard was more influenced by outlier observations than was CDC ILI Surveillance data.

Most of the influential observations occurred during the peak 2003–04 influenza season. This season was characterized by early and intense influenza activity, a large number of influenza-associated pediatric deaths, and increased media attention to influenza [18]. It is possible that during this influenza season, physician laboratory testing patterns or patient health care seeking behavior differentially affected the relationship between ILI rates and laboratory confirmation of influenza. Additionally, internet search behavior about respiratory infections during this period could have been different than during subsequent, more typical influenza seasons. These findings are relevant to the applicability of surveillance using internet key word searches during the 2009 H1N1 pandemic and future anomalous influenza seasons.

The Google Flu Trends statistical model was created and validated using rates of ILI, which is a nonspecific syndrome that is not necessarily caused by influenza virus infection, but used for decades as an indicator of the burden of outpatient influenza illness. Any nationwide surveillance using internet key word search is likely to be most representative if the search engines being monitored has widespread use. As the popularity of a particular internet search engine wanes, so too may the overall accuracy of disease activity estimates using its data. Also, the stability of internet key word surveillance relies on consistency of internet search behavior [19], as well as search term use between geographic regions and over time. Changing media trends, word search choices, and cultural make-up of regions and over time may also affect the representativeness of internet search surveillance.

Our analyses represent the first comparison of Google Flu Trends data with data on laboratory-confirmed influenza virus infections. Prior studies have demonstrated that Google Flu Trends can estimate rates of nonspecific ILI in New Zealand, Europe, and the United States [2], [20], [21]. We have demonstrated that Google Flu Trends performs less well when estimating surveillance data for laboratory-confirmed influenza, which is not surprising, as the Google Flu Trends algorithm was developed using only ILI data.

There are several US influenza surveillance systems [10], and taken as a whole, they provide an excellent overview of influenza activity at any period during the influenza season. However, only CDC Virus Surveillance data tracks nationwide activity of laboratory-confirmed influenza. The original publication describing and validating the Google Flu Trends methods intentionally excluded specifics concerning the statistical model used out of concern that public knowledge of the search terms could alter its usefulness to track influenza activity [2]. Nevertheless, without the publication of the Google Flu Trends statistical model, further independent, prospective validation or improvements upon the model are not possible.

This study is subject to limitations. While US Influenza Virologic Surveillance System provides the best data source for following trends in laboratory-confirmed influenza infections, it is nevertheless a convenience sample of specimens sent to participating laboratories. In addition, health care seeking behavior, physician testing practices, and internet search behavior may change over time or through the course of an influenza epidemic, limiting the interpretation of correlation data from this analysis.

In conclusion, Google Flu Trends may make a useful contribution to public health given the timeliness of the data and its close association with traditional US ILI surveillance system data. However, CDC ILI Surveillance and positive influenza tests were more correlated during the five years of this study, including the unusual 2003–04 influenza season, than were Google Flu Trends and positive influenza tests. We hypothesize that differences in internet search behavior, patient health care seeking behavior, and physician testing practices may alter the correlation between influenza surveillance systems. In the absence of influenza virologic surveillance, sentinel surveillance for ILI may more accurately monitor influenza activity than Google Flu Trends during anomalous influenza seasons. Furthermore, given the non-specific nature of ILI, robust nationwide virologic surveillance remains critical to the understanding of influenza activity during inter-pandemic and pandemic periods alike.

## Supporting Information

Table S1 **Pearson**'**s Correlation Coefficient Matrix of Data from Three Influenza Surveillance Systems including CDC Influenza Virologic Surveillance with One and Two Week Lag, by Influenza Season September 28, 2003 through May 17, 2008^1^.** Note: ^1^Because CDC surveillance is intensified from calendar week 40 through calendar week 20 of the subsequent year, we restricted our correlation analyses to this time period.
(DOC)

Table S2 **Pearson**'**s Correlation Coefficient Matrix of Data from Three Influenza Surveillance Systems including CDC Influenza Virologic Surveillance with One and Two Week Lag, by US Census Region September 28, 2003 through May 17, 2008^1^.** Note: 1. Because CDC surveillance is intensified from calendar week 40 through calendar week 20 of the subsequent year, we restricted our correlation analyses to this time period.
(DOC)

Table S3 **Calendar Week of Peak Influenza Activity per Influenza Surveillance Year for Three Influenza Surveillance Systems: Google Flu Trends, CDC Influenza-like Illness Surveillance, and CDC Influenza Virologic Surveillance, September 28, 2003 through May 17, 2008.**
(DOC)

Table S4 **Mean of US Census Region^1^ Pearson**'**s Correlation Coefficients by Influenza Season of Data from Three Influenza Surveillance Systems: Google Flu Trends, CDC Influenza-like Illness Surveillance, CDC Influenza Virologic Surveillance, September 28, 2003 through May 17, 2008^2^. **Note: 1. US Census Regions include the following states: (1) New England; Connecticut, Maine, Massachusetts, New Hampshire, Rhode Island, Vermont; (2) Middle Atlantic; New Jersey, New York, Pennsylvania; (3) East North Central; Indiana, Illinois, Michigan, Ohio, Wisconsin; (4) West North Central; Iowa, Kansas, Minnesota, Missouri, Nebraska, North Dakota, South Dakota; (5) South Atlantic; Delaware, District of Columbia, Florida, Georgia, Maryland, North Carolina, South Carolina, Virginia, West Virginia; (6) East South Central; Alabama, Kentucky, Mississippi, Tennessee; (7) West South Central; Arkansas, Louisiana, Oklahoma, Texas; (8) Mountain; Arizona, Colorado, Idaho, New Mexico, Montana, Utah, Nevada, Wyoming; (9) Pacific; Alaska, California, Hawaii, Oregon, Washington. 2. Because CDC surveillance is intensified from calendar week 40 through calendar week 20 of the subsequent year, we restricted our correlation analyses to this time period.
(DOC)
