# Supplementary material for: Monitoring Influenza Activity in the United States: A Comparison of Traditional Surveillance Systems with Google Flu Trends
Source: PLoS One. 2011 Apr 27;6(4):e18687. doi: 10.1371/journal.pone.0018687 (PMC3083406; doi:10.1371/journal.pone.0018687)
Supplement: Table S1 — Pearson's Correlation Coefficient Matrix of Data from Three Influenza Surveillance Systems including CDC Influenza Virologic Surveillance with One and Two Week Lag, by Influenza Season September 28, 2003 through May 17, 20081. Note: 1Because CDC surveillance is intensified from calendar week 40 through calendar week 20 of the subsequent year, we restricted our correlation analyses to this time period. (DOC) [file pone.0018687.s001.doc]

**Supplemental Table S1. Pearson’s Correlation Coefficient Matrix of Data from Three Influenza Surveillance Systems including CDC Influenza Virologic Surveillance with One and Two Week Lag, by Influenza Season September 28, 2003 through May 17, 2008^1^**

| Surveillance Year | Dataset | Google Flu Trends | CDC ILI |
| --- | --- | --- | --- |
| 2003-04 | Google Flu Trends | 1.00 | -- |
|  | CDC ILI | 0.95 | 1.00 |
|  | CDC Virologic | 0.67 | 0.81 |
|  | CDC Virologic -- One Week Lag | 0.77 | 0.89 |
|  | CDC Virologic -- Two Week Lag | 0.82 | 0.92 |
| 2004-05 | Google Flu Trends | 1.00 | -- |
|  | CDC ILI | 0.98 | 1.00 |
|  | CDC Virologic | 0.94 | 0.94 |
|  | CDC Virologic -- One Week Lag | 0.93 | 0.94 |
|  | CDC Virologic -- Two Week Lag | 0.85 | 0.88 |
| 2005-06 | Google Flu Trends | 1.00 | -- |
|  | CDC ILI | 0.97 | 1.00 |
|  | CDC Virologic | 0.72 | 0.82 |
|  | CDC Virologic -- One Week Lag | 0.62 | 0.72 |
|  | CDC Virologic -- Two Week Lag | 0.48 | 0.58 |
| 2006-07 | Google Flu Trends | 1.00 | -- |
|  | CDC ILI | 0.94 | 1.00 |
|  | CDC Virologic | 0.71 | 0.79 |
|  | CDC Virologic -- One Week Lag | 0.64 | 0.70 |
|  | CDC Virologic -- Two Week Lag | 0.50 | 0.55 |
| 2007-08 | Google Flu Trends | 1.00 | -- |
|  | CDC ILI | 0.98 | 1.00 |
|  | CDC Virologic | 0.91 | 0.92 |
|  | CDC Virologic -- One Week Lag | 0.86 | 0.86 |
|  | CDC Virologic -- Two Week Lag | 0.75 | 0.74 |

Note:

1. Because CDC surveillance is intensified from calendar week 40 through calendar week 20 of the subsequent year, we restricted our correlation analyses to this time period.
