# Supplementary material for: Monitoring Influenza Activity in the United States: A Comparison of Traditional Surveillance Systems with Google Flu Trends
Source: PLoS One. 2011 Apr 27;6(4):e18687. doi: 10.1371/journal.pone.0018687 (PMC3083406; doi:10.1371/journal.pone.0018687)
Supplement: Table S3 — Calendar Week of Peak Influenza Activity per Influenza Surveillance Year for Three Influenza Surveillance Systems: Google Flu Trends, CDC Influenza-like Illness Surveillance, and CDC Influenza Virologic Surveillance, September 28, 2003 through May 17, 2008. (DOC) [file pone.0018687.s003.doc]

**Supplemental Table S3. Calendar Week of Peak Influenza Activity per Influenza Surveillance Year for Three Influenza Surveillance Systems: Google Flu Trends, CDC Influenza-like Illness Surveillance, and** CDC Influenza Virologic Surveillance, September 28, 2003 through May 17, 2008

| Season | Google Flu Trends | CDC ILI Surveillance | CDC Virologic Surveillance |
| --- | --- | --- | --- |
| 2003-04 | 50 | 52 | 48 |
| 2004-05 | 6 | 7 | 5 |
| 2005-06 | 52 | 53 | 9 |
| 2006-07 | 1 | 7 | 6 |
| 2007-08 | 8 | 7 | 7 |
