# Supplementary material for: Monitoring Influenza Activity in the United States: A Comparison of Traditional Surveillance Systems with Google Flu Trends
Source: PLoS One. 2011 Apr 27;6(4):e18687. doi: 10.1371/journal.pone.0018687 (PMC3083406; doi:10.1371/journal.pone.0018687)
Supplement: Table S4 — Mean of US Census Region1 Pearson's Correlation Coefficients by Influenza Season of Data from Three Influenza Surveillance Systems: Google Flu Trends, CDC Influenza-like Illness Surveillance, CDC Influenza Virologic Surveillance, September 28, 2003 through May 17, 20082. Note: 1. US Census Regions include the following states: (1) New England; Connecticut, Maine, Massachusetts, New Hampshire, Rhode Island, Vermont; (2) Middle Atlantic; New Jersey, New York, Pennsylvania; (3) East North Central; Indiana, Illinois, Michigan, Ohio, Wisconsin; (4) West North Central; Iowa, Kansas, Minnesota, Missouri, Nebraska, North Dakota, South Dakota; (5) South Atlantic; Delaware, District of Columbia, Florida, Georgia, Maryland, North Carolina, South Carolina, Virginia, West Virginia; (6) East South Central; Alabama, Kentucky, Mississippi, Tennessee; (7) West South Central; Arkansas, Louisiana, Oklahoma, Texas; (8) Mountain; Arizona, Colorado, Idaho, New Mexico, Montana, Utah, Nevada, Wyoming; (9) Pacific; Alaska, California, Hawaii, Oregon, Washington. 2. Because CDC surveillance is intensified from calendar week 40 through calendar week 20 of the subsequent year, we restricted our correlation analyses to this time period. (DOC) [file pone.0018687.s004.doc]

**Supplemental Table S4. Mean of US Census Region^1^ Pearson’s Correlation Coefficients by Influenza Season of Data from Three Influenza Surveillance Systems: Google Flu Trends, CDC Influenza-like Illness Surveillance, CDC Influenza Virologic Surveillance, September 28, 2003 through May 17, 2008^2^**

|  | Google Flu Trends – CDC ILI Surveillance | Google Flu Trends – CDC Virologic Surveillance | CDC ILI – CDC Virologic Surveillance |
| --- | --- | --- | --- |
| Season | Mean Correlation (SD) | Mean Correlation (SD) | Mean Correlation (SD) |
| 2003-04 | 0.94 (0.04) | 0.79 (0.10) | 0.86 (0.07) |
| 2004-05 | 0.95 (0.02) | 0.88 (0.07) | 0.88 (0.06) |
| 2005-06 | 0.87 (0.08) | 0.70 (0.09) | 0.73 (0.20) |
| 2006-07 | 0.87 (0.10) | 0.63 (0.23) | 0.68 (0.28) |
| 2007-08 | 0.97 (0.02) | 0.87 (0.04) | 0.89 (0.05) |

Note:

1. US Census Regions include the following states: (1) New England; Connecticut, Maine, Massachusetts, New Hampshire, Rhode Island, Vermont; (2) Middle Atlantic; New Jersey, New York, Pennsylvania; (3) East North Central; Indiana, Illinois, Michigan, Ohio, Wisconsin; (4) West North Central; Iowa, Kansas, Minnesota, Missouri, Nebraska, North Dakota, South Dakota; (5) South Atlantic; Delaware, District of Columbia, Florida, Georgia, Maryland, North Carolina, South Carolina, Virginia, West Virginia; (6) East South Central; Alabama, Kentucky, Mississippi, Tennessee; (7) West South Central; Arkansas, Louisiana, Oklahoma, Texas; (8) Mountain; Arizona, Colorado, Idaho, New Mexico, Montana, Utah, Nevada, Wyoming; (9) Pacific; Alaska, California, Hawaii, Oregon, Washington.

2. Because CDC surveillance is intensified from calendar week 40 through calendar week 20 of the subsequent year, we restricted our correlation analyses to this time period.
